# Improving the hydrophilic microenvironment surrounding the catalytic site of fructosyltransferase enhances its catalytic ability

**DOI:** 10.1007/s10529-025-03566-8

**Published:** 2025-02-26

**Authors:** Fanzhi Wang, Suren Singh, Kugen Permaul

**Affiliations:** Department of Biotechnology and Food Science, Durban University of Technology, Durban, 4001 South Africa

**Keywords:** Catalytic ability, Fructosyltransferase, Fructooligosaccharides, Hydrophilic microenvironment

## Abstract

The hydrophilic microenvironment surrounding an enzyme's active site can influence its catalytic activity. This study examines the effect of enhancing this environment in the *Aspergillus niger* fructosyltransferase, SucC. Bioinformatics analysis identified a cysteine residue (C66) near the catalytic triad (D64, D194, E271) as vital for maintaining the active site's structure and facilitating substrate transport. Simulated mutagenesis suggested that mutating cysteine to serine (C66S) could increase hydrophilicity without altering the structure significantly. This mutation was predicted to enhance substrate affinity, with binding energy changing from −3.65 to −4.14 kcal mol^−1^. The C66S mutant, expressed in *Pichia pastoris* GS115, showed a 61.3% increase in specific activity, a 13.5% decrease in *K*_m_ (82.20/71.14 mM), and a 21.6% increase in *k*_cat_ (112.23/136.48 min^−1^), resulting in a 40.1% increase in catalytic efficiency (1.37/1.92 min^−1^ mM^−1^). For fructooligosaccharides (FOS) production, C66S demonstrated enhanced transfructosylation, particularly in the initial stages of the reaction, achieving higher overall FOS yields. These findings highlight that modifying the active site hydrophilicity, without causing major structural changes, is a promising strategy for improving an enzyme’s catalytic efficiency.

**Supplementary Information:**

The online version contains supplementary material available at 10.1007/s10529-025-03566-8.

## Introduction

Enzymes are remarkable biocatalysts, essential for life and ubiquitous in biotechnological applications. These complex molecules are synthesized intracellularly via. transcription and translation, representing the expression of specific genes (Singh et al. 2019). Enzymes are indispensable in various industries, including the production of biofuels, pharmaceuticals, and specialty chemicals (Mesbah 2022). In the food industry, they contribute to the manufacture of prebiotics, alcohols, and dairy products (Raveendran et al. 2018), while in medical diagnostics, they are pivotal in tests and assays (Singh et al. 2019). Enzymes also play roles in environmental bioremediation (Bhandari et al. 2021) and the textile industry, where they are used for desizing and biofinishing (Madhu and Chakraborty 2017). The demand for efficient and stable enzymes is ever-growing, leading to extensive screening and discovery efforts (Madhavan et al. 2021; Zhu et al. 2022) and the advancement of enzyme engineering through artificial evolution techniques (Sharma et al. 2021). Recently, computational advancements have allowed profound insights into the structure–function dynamics of enzyme active sites, facilitating the creation of enzymes with improved industrial applicability (Chowdhury and Maranas 2020; Madhavan et al. 2021; Miller et al. 2022).

The intricacies of enzyme catalysis have been explored through various models. Traditional theories such as the lock-and-key (Fischer 1894), induced-fit (Koshland 1958), and keyhole–lock–key models (Prokop et al. 2012) describe substrate interactions and the catalytic process. A deeper understanding of the microenvironment surrounding an enzyme's active site is essential for both fundamental research and practical applications (Buller et al. 2023). Factors like microenvironment volume (Ma et al. 2024), hydrophilicity/hydrophobicity (Yang et al. 2022), pH balance (Lu et al. 2002), and electrostatic charges (Meiering et al. 1992) all play crucial roles. Among these, the hydrophilic and hydrophobic balance close to the active site is particularly significant. Hydrophilic residues in substrate tunnels can enhance substrate binding and transport, impacting overall enzymatic efficiency (Kaushik et al. 2018; Singh and Anand 2021). Considering the aqueous nature of enzymatic environments, it is suggested that the hydrophilic/hydrophobic microenvironment can influence catalytic activity (Wang et al. 2021a, b; Yang et al. 2022). However, the impact of increased active site hydrophilicity on enzyme activity has not been explicitly reported.

This study hypothesizes that enhancing the hydrophilic microenvironment of the active site can significantly improve the catalytic efficiency of fructosyltransferases, based on the principle that hydrophilic sucrose exhibits strong affinity to hydrophilic residues. *Aspergillus niger* fructosyltransferase, SucC, was selected as the model enzyme for fructooligosaccharides (FOS) production. The research involves a detailed illustration of the superstructure surrounding the active site and substrate tunnel, identifying and selecting candidate amino acid residues that could influence catalytic activity. The potential impacts of these residues on the enzyme's structure and active site hydrophilicity are predicted and analyzed. Subsequently, candidate mutants were constructed through site-directed mutagenesis, expressed in a heterologous host, and their biochemical properties examined. These findings emphasize that modifying active site hydrophilicity, without major structural changes, is a promising strategy for enhancing enzyme catalytic efficiency and broadening their industrial applications.

## Materials and methods

### Structure prediction

SucC (AHC54391.1) was modelled using the SWISS-MODEL server (https://swissmodel.expasy.org/). Fructofuranosidase 5XH8 (Nagaya et al. 2017) was used as the existing template for modelling of SucC. The output results showed sequence similarities and GMQE scores of each generated structure where GMQE stands for “Global Model Quality Estimation” and is a score used to estimate the quality of the 3-D model generated by Swiss-Model. The GMQE score was expressed as a value between 0 and 1 where a higher score indicates a better model of higher quality (Waterhouse et al. 2018). The QMEANDisCo value (Qualitative Model Energy Analysis Discrimination Score) Global was also generated and provided a measure of the overall reliability of the protein structure model (Studer et al. 2020). The structure produced from SWISS-MODEL was then rendered or visualized using PyMOL (Schrodinger 2015).

The amino acid sequence and primary structure alignments to determine conserved sequences surrounding active sites was carried out using ClustalW (Thompson et al. 1994). The amino acid sequences of three fructosyltransferases: two invertases and one fructofuranosidase from different microbial sources which had their catalytic residues identified (Olarte-Avellaneda et al. 2018) and have been used for synthesizing FOSs, were selected as the reference sequences.

### Access tunnel analysis

The access tunnels in SucC were simulated by the Caver 3.0.3 Plugin in PyMOL (Chovancova et al. 2012). The tertiary structures of SucC and its mutants were predicted and compared for protein structure analysis. The starting point was set in the centre of the catalytic triad in SucC. The minimum probe radius was set to 0.7 Å and the shell depth and shell radius were set to 4 and 5 Å, respectively. The clustering threshold was set to 3.5 Å, the maximum distance was set to 3 Å and the desired radius was set to 5 Å.

### Screening and selection of mutants

Autodock 1.5.7 (Morris et al. 2009) was used where a Lamarckian genetic algorithm was specifically applied to model the interaction of sucrose with SucC by predicting the most energetically favourable binding mode of sucrose within the active site of SucC. Dockings were run 60 times and sucrose binding was restricted to the catalytic region. The amino acid residues interacting with sucrose in the active site were initially determined, and mutations were subsequently designed to modify the catalytic regions. The tertiary structures of SucC and its mutant candidates were predicted using SWISS-MODEL (Waterhouse et al. 2018) using the most related model enzyme, the fructofuranosidase from *Aspergillus kawachii* (PDB ID: 5XH8) (Nagaya et al. 2017). The interactions between proposed mutants and sucrose were predicted by Autodock (Morris et al. 2009) and visualized by PyMOL (Schrodinger 2015).

### Site-directed mutagenesis, molecular cloning and expression

The SucC mutants were created by site-directed mutagenesis using the over-lapping PCR technique (Hussain and Chong 2016). Primers were designed to obtain overlapping fragments where codons for the target amino acid were deliberately replaced by codons of the mutated amino acid. The designed primers are listed in Table 1. The obtained up-stream and down-stream overlapping fragments were then hybridized to form complete genes which were then cloned into plasmid pPIC9K (Invitrogen) for protein expression.

**Table 1:** Primers used to create the C66S mutant

Primers	Oligonucleotide Sequence (5΄ to 3΄)*
SucC-F	GTAAAGCTTCAAACGGCTTCCG
SucC-R	TGCTCTAGATTAAGACTGACGATCCGGCC
C66S-F	CAGATCGGTGACCCC***TCT***CTGCATTACACCGATCCT
C66S-R	CAG***AGA***GGGGTCACCGATCTG

*The underlined sequence represents the *Xba*I recognized restriction site. The bold and italicized sequences symbolize the mutated region

The gene encoding SucC was codon-optimized and chemically synthesized. The *sucC* mutants were cloned downstream of the α-factor in pPIC9K to ensure functional expression in *Pichia pastoris* GS115 (Invitrogen), as described by the manufacturer’s guidelines. The sequences of mutants were verified by DNA sequencing using the ABI 3730*xl* Genetic Analyzer (Applied Biosystems, Thermo Fisher Scientific). All cloning procedures were carried out following the standard molecular cloning processes (Green and Sambrook 2012). The recombinant *P. pastoris* GS115 strains were recovered on YPD [1% yeast extract (w/v); 2% peptone (w/v); 2% glucose (w/v)] agar plates containing 2 mg geneticin mL^−1^ at 30 °C and 220 rpm for 72 h. Shake flask fermentations at 50 mL scale were performed to express the enzymes, where one colony was subcultured into 20 mL liquid YPD medium and incubated at 30 °C and 200 rpm for 16 h. The 250 μL culture suspension was inoculated into 25 mL BMGY [1% yeast extract (w/ v); 2% peptone (w/v); 2% glucose (w/v); 1 M potassium phosphate buffer (pH 6.0 ± 0.2; 1% glycerol (w/v); 4 × 10^–5^% biotin (w/v); 1.34% yeast nitrogen base (Oxoid) (w/v)] medium to represent a 1% inoculum and then incubated at 30℃ and 200 rpm until the cell suspension reached an OD_600_ of between 4 to 5. Cells were then collected by centrifugation at 6000 × *g* and 4 °C for 5 min and resuspended by 2 mL BMMY [1% yeast extract (w/ v); 2% peptone (w/v); 2% glucose (w/v); 1 M potassium phosphate buffer (pH 6.0 ± 0.2; 1% glycerol (w/v); 4 × 10^–5^% biotin (w/v); 1.34% yeast nitrogen base (w/v)] medium. The fermentation medium was inoculated with the cell suspension to reach an OD_600_ of 1.0 at the start of the fermentation. The fermentation was supplemented with 250 μL methanol (0.5%, v/v) as the inducer, every 24 h and the fermentation lasted up to 120 h at 30 °C and 200 rpm. The supernatants were collected as the crude enzymes for characterization.

### Enzyme purification

The recovered enzyme-containing supernatant was desalted using a PD-10 Desalting Column (GE Inc.) and purified in an AKTA 100 purification system (Cytiva, Sweden). A Superdex 200 Increase 10/300 GL (Cytiva, Sweden) gel filtration column was used for size exclusion chromatography purification of proteins according to the manufacturer’s guideline. The column as well as the purification system was equilibrated by the equilibration buffer (10 mM phosphate, 150 mM NaCl, pH 7.4). After equilibration, 500 μL samples were loaded. The elution rate was set to 1 mL min^−1^. All protein fractions were collected in 300 μL aliquots. The collected fractions were analysed by detecting their enzyme activity by enzyme assays. The SDS-PAGE technique (Laemmli 1970) was to verify the purity of SucC and its mutants.

### Enzyme activity assay

The activities of SucC and its mutants were determined by measuring the amount of glucose generated in the enzymatic reaction, which reflects the transfructosylating activity, using a glucose detector (Yoo and Lee 2010). This approach is based on the prior confirmation that the enzyme used in this study exclusively exhibits transfructosylation activity, forming fructooligosaccharides (FOS) while releasing glucose, without hydrolyzing sucrose into glucose and fructose. The enzyme reaction was conducted in a 10% (m/v) sucrose solution in 100 mM sodium phosphate buffer at 50 °C and pH 5.5 for 1 h. The reaction was terminated by boiling for 10 min. One unit (U) of enzyme activity was defined as the amount of enzyme required to transfer 1 μmol of fructose, corresponding to the amount of glucose released, per min at 50 °C and pH 5.5. The protein concentrations of purified SucC and its mutants were quantified using the Bradford method (Bradford 1976). The specific activity was subsequently determined as the ratio of enzyme activity to the pure protein concentration.

### Impact of temperature and pH on enzyme activity and stability

The temperature and pH optima were determined using the standard enzyme activity assay at different temperatures (30–80 °C) and constant pH (5.5); at different pH (4–9) and constant temperature (50 °C), respectively. The buffers (100 mM) used were: citric acid buffer (pH 4.0–6.0); phosphate buffer (pH 6.0–8.0); and Tris/HCl (pH 8.0–9.0). The thermostability and pH stability were assayed by incubating SucC and its mutants at different temperatures (4–80 °C) and pH (4–9) for 1 h, respectively. The remaining activities of these incubated enzymes were then determined by the standard enzyme activity assay.

### Determination of kinetic parameters

The pure enzymes were used to determine the *K*_m_, *V*_max_ and *k*_cat_ values. A series of sucrose solutions were prepared with different concentrations (50, 80, 110, 150, 200, 250 mM). The reaction mixtures were composed of 900 μL substrate and 100 μL enzyme solution where the amount of enzyme added into the reaction mixtures was adjusted to a final activity of 5 U mL^−1^ in 1 mL reaction mixture by dilutions prior to the reactions for determination of enzyme kinetics. The reactions were carried out at 50 °C and pH 5.5 for 5 min and terminated by incubation at 80 °C for 10 min. The generated glucose was then detected by a biosensor. Origin 9 (OriginLab) software was used to analyze the collected data and enzyme kinetics were then determined through its Nonlinear Fitting mode that fits the Michaelis–Menten function. The values of *K*_m_, *V*_max_ and *k*_cat_ for enzymes being analyzed were then determined.

### FOS generation and sugar profiles analyzed by HPLC

Enzyme reactions for preparation of FOS from sucrose were performed in a 100 mL reaction mixture with 400 g sucrose L^−1^ as the substrate. The quantity of enzyme added into the different reactions were standardized to 9 U per 1 g sucrose and the reactions were optimized at 50 °C and pH 5.5 in a water bath. Six hours reactions were performed where 1 mL of the reaction mixture was collected every hour, and the reaction terminated by incubating at 80 °C for 10 min. The collected reaction samples were analyzed by HPLC using the Agilent 1200 High Performance Liquid Chromatography System (Agilent Technologies). The Prevail Carbohydrate ES HPLC column (5.0 μm, 250 × 4.6 mm) was equilibrated and eluted with 65% (v/v) acetonitrile solution with a 1 mL min^−1^ flow rate at 35 °C. The Alltech ELSD 2000 (Grace Davison Discovery Sciences) was used as the detector. Glucose and FOS standards were purchased from Sigma Aldrich. The data obtained was analyzed and the concentrations of different FOS components were calculated based on peak areas from a calibration curve generated from glucose and FOS standard solutions.

### Statistical analysis

All experiments were carried out in triplicate and data was presented as mean ± standard deviation (SD). Statistical significance was determined by one-way ANOVA, followed by Tukey's post-hoc test. Statistical analyses were performed using GraphPad Prism 8.0.2 (GraphPad Software) and differences were considered significant at *P* < 0.05.

## Results and discussion

### Structural characteristics of* A. niger* SucC

**Fig. 1:**
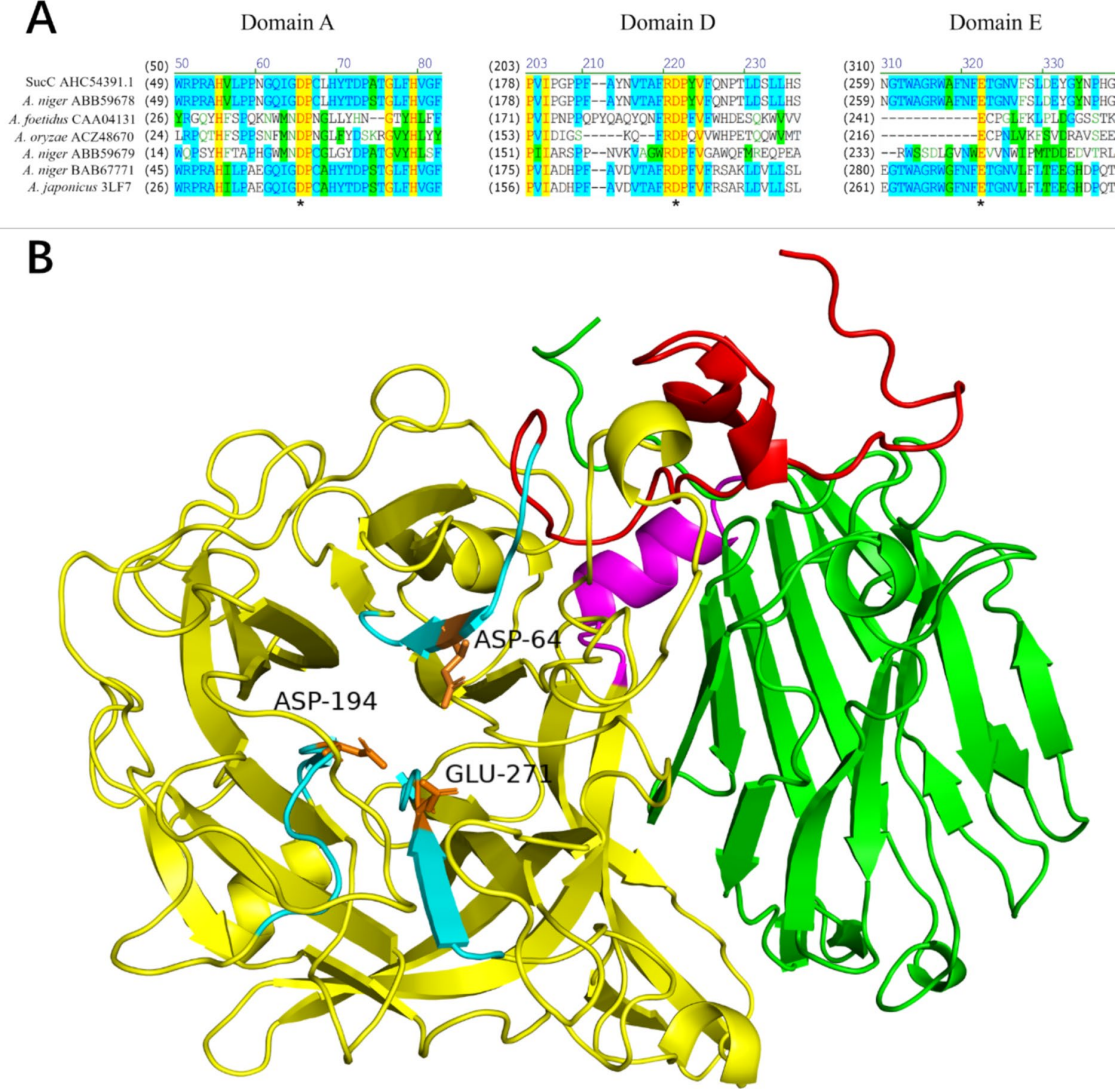
Sequence alignment and structural analysis of SucC. **A** Primary sequence alignment of SucC with the other FOS-generating enzymes (Olarte-Avellaneda et al. 2018). The active site was predicted where D64 in domain A, D194 in domain D and E271 in domain E were identified as the catalytic triad (marked with asterisks). Yellow identifies the identical residues, green represents blocks of similar residues, cyan represents conserved residues and non-coloured residues are non-similar. The selected reference sequences included: a fructosyltransferase from *A. foetidus* (GenBank accession number: CAA04131); a fructosyltransferase from *A. oryzae* N74 (ACZ48670); an intracellular invertase from *A. niger* (ABB59679); an extracellular invertase from *A. niger* (ABB59678); a fructosyltransferase from *A. japonicus* (PDB ID: 3LF7); and a β-fructofuranidase from *A. japonicus* ATCC (BAB67771). **B** Predicted tertiary structure of SucC. The SucC tertiary structure was predicted through PyMOL where the enzyme was modelled by SWISS-MODEL, with the fructofuranosidase, 5XH8 used as template. The components of all regions were determined and presented in different colours. Red represents an N-terminal small component (25–59), yellow represents the β-propeller domain (60–440), magenta represents the α-helical linker (residues 441–452), and green represents the C-terminal β-sandwich domain (453–628). Three highly conserved domains are coloured cyan with Asp-64, Asp-194, Glu-271 (orange) being the active sites, forming a catalytic triad

The amino acid sequence alignment between fructosyltransferase SucC from *A. niger* and six other FOS-generating enzymes with identified catalytic residues was made and the catalytic residues in SucC were predicted and characterized (Fig. 1a). Although the amino acid sequences from different sources were quite distinct to each other, the typical two Asp and one Glu in the highly conserved domains were 100% identical at the sites D64, D194 and E271 in SucC where one Asp (D64) in Domain A was the catalytic nucleophile, the other Asp (D194) in Domain D was a transition stabilizer, and Glu (E271) in Domain E was the general acid/base catalyst. They together formed a catalytic triad that was responsible for catalysis (Fig. 1a and b) in which the catalytic triad formed with two Asp and one Glu residue. This is a typical and unique characteristic among the GH32 and GH68 family glycoside hydrolases (Sainz-Polo et al. 2013; Olarte-Avellaneda et al. 2018; Manoochehri et al. 2020).

SucC was further modeled by SWISS-MODELING using the crystal structure of a *Aspergillus kawachii* fructofuranosidase (PDB ID: 5XH8) (Nagaya et al. 2017) as template which was the most closely related model to SucC (Fig. 1b). The GMQE value of the generated SucC tertiary structure was 0.91 and the QMEANDisCo Global score was 0.88 ± 0.05, which indicated a reliable model was generated with high quality. The 3-D structure was composed of: an N-terminal small component (residues 25–59, component NS); a β-propeller domain (residues 60–440); an α-helical linker (residues 441–452); and a C-terminal β-sandwich domain (residues 453–628). Specifically, the five-fold β-propeller catalytic domain was present with Asp-64, Asp-194, Glu-271 being the active sites forming a catalytic triad (Fig. 1a and b). In summary, SucC from *A. niger* belonged to the GH32 family as it shared a common characteristic among GH32 enzymes (invertases, inulinases and fructosyltransferases) that contained an additional β-sandwich structure which was attached to the five-fold β-propeller domain (Lammens et al. 2009). The identification of the acidic catalytic triad (D64, D194 and E271) in the three highly conserved motifs (Domain A, Domain D and Domain E) within the five-fold β-propeller domain was a typical and unique characteristic among family GH32 (Pons et al. 2004; Yuan et al. 2006; Lammens et al. 2009; Alméciga-Díaz et al. 2011). The bioinformatics data showed that a reliable SucC 3D structure was produced which served as the basis for further analysis.

### Selection of SucC mutant candidates by bioinformatics analysis

**Fig. 2:**
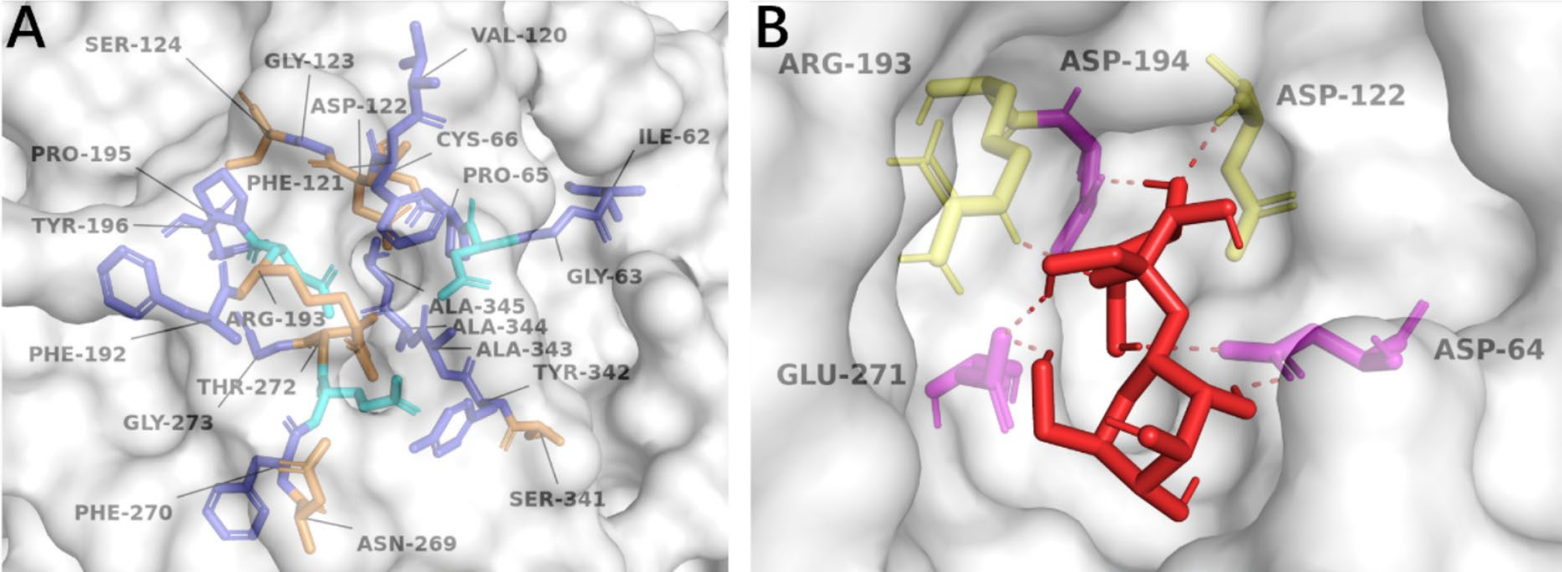
The micro-environment of SucC surrounding the active site. **A** The amino acid residues surrounding the active site. The hydrophobic active site where the catalytic triad was labelled cyan, the hydrophobic residues were labelled blue and hydrophilic residues were labelled orange. **B** The binding model of sucrose to SucC. The substrate sucrose is coloured red, the interacting residues are composed of the catalytic triad (Asp-64. Asp-194 and Glu-271) (magenta) and Arg-122, Asp-193 (yellow)

The strategy for selecting specific amino acids for mutation was to increase the hydrophilicity of the enzyme's catalytic regions while minimizing any alterations to the active site's conformation. Given that sucrose contains multiple hydroxyl groups and tends to interact favorably with a hydrophilic environment, the microenvironment around the catalytic triad of SucC was further analyzed, as depicted in Fig. 2A. The microenvironment surrounding the catalytic triad (D64, D194, and E271) was predominantly hydrophobic, consisting of residues such as Ile-62, Gly-63, Pro-65, Val-120, Phe-121, Gly-123, Phe-192, Pro-195, Tyr-196, Phe-270, Gly-273, Tyr-342, Ala-333, Ala-344, and Ala-345. Meanwhile, the hydrophilic residues in proximity to the catalytic triad included Cys-66, Asp-122, Ser-124, Arg-193, Asn-269, Thr-272, and Ser-341. These residues were vital for maintaining the conformation of the active site.

The substitution of an amino acid residue near the catalytic triad can potentially lead to significant changes in enzyme performance (Coulther et al. 2021). As shown in Fig. 2b, residues such as Asp-64, Asp-122, Arg-193, Asp-194, and Glu-271 were not considered for substitution because they directly interact with sucrose. Similarly, Pro-65, Phe-121, Gly-123, Phe-192, and Pro-195 were not targeted due to their high conservation and essential roles in catalysis. The hydrophilic residues Asp-122, Ser-124, Arg-193, Asn-269, Thr-272, and Ser-341 were also not considered for substitution in this study. Among other candidates, Ile-62, Val-120, and Phe-270 were not considered for mutagenesis due to their strong hydrophobicity, as their substitution might destabilize the active site structure, thus impacting the catalytic function. Gly-273 was left unchanged because its simple side-chain structure plays a role in maintaining the conformation of the substrate access tunnel; altering this residue might obstruct substrate or product transport. Lastly, Tyr-342 was not considered for substitution due to its -OH group, which could participate in substrate binding via hydrogen bonds. The aromatic ring and hydroxyl group of Tyr likely facilitate specific substrate recognition, making this residue crucial for catalysis.

Surprisingly, only one cysteine (Cys) residue is present in the active site microenvironment, exhibiting both hydrophobic and polar traits. Chemically, cysteine contains sulfur, which contributes to the stability of the active site due to its weak polarity (Iyer and Mahalakshmi 2019). Cys-66 did not directly interact with the substrate (Fig. 2b), but it was deduced that Cys-66 primarily plays a role in maintaining and stabilizing the tertiary structure of the substrate access tunnel. Therefore, Cys-66 was selected for a targeted saturated mutagenesis analysis. As summarized in Supplementary Table S1, variant enzymes with Cys-66 substituted by any of the other 19 possible amino acids all showed increased affinity for sucrose compared to WT-SucC (−3.65 kcal mol^−1^). The C66S variant exhibited the most significant improvement in affinity to sucrose at −4.14 kcal mol^−1^. While Asp-64, Asp-122, Arg-193, Asp-194, and Glu-271 were predicted to directly interact with sucrose, C66S was found to have two additional interacting residues, Glu-296 and His-310. This finding suggests that C66S positively modified the catalytic region's microenvironment, making the substrate more accessible to the active site than in the wild-type SucC and other predicted mutants. Given the very similar conformational structures of cysteine and serine differing only in their side chains (–SH in Cys vs. –OH in Ser), it was predicted that substituting Cys with Ser would not lead to significant changes in the active site's conformation, but could enhance the hydrophilicity of the active site. This change should favor interactions between sucrose and the active site through hydrogen bonds, due to the presence of multiple hydroxyl groups. Thus, the C66S mutation was selected for further study.

### Sucrose access tunnel simulations

**Fig. 3:**
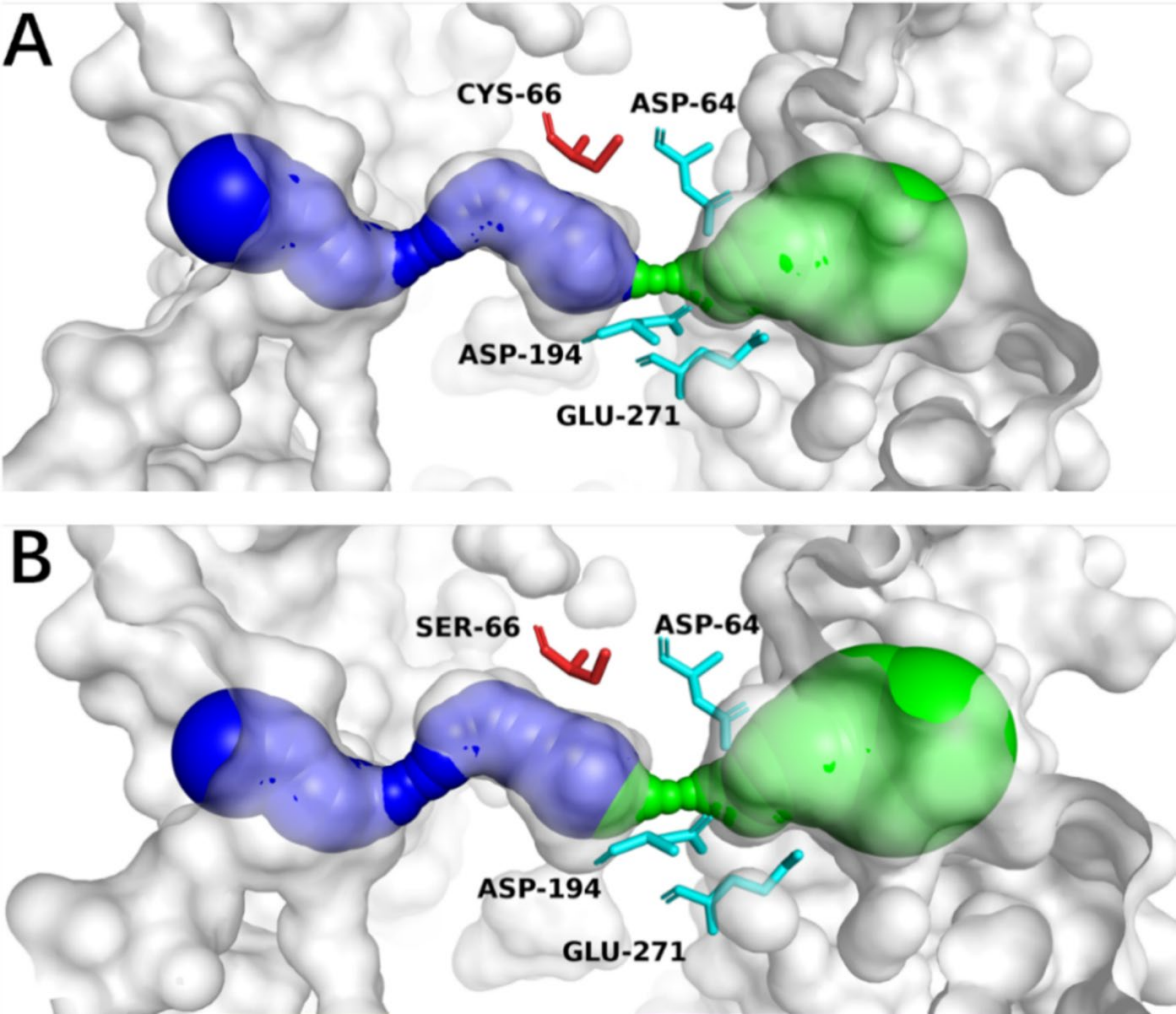
Structure analysis of wild type SucC (**a**) and SucC-C66S (**b**) depicting the modelled tunnels T1 (green) and T2 (blue). The catalytic triad (D64, D194 and E271) were colored in cyan while Cys-66 (**a**) and Ser-66 (**b**) were colored in red

**Fig. 4:**
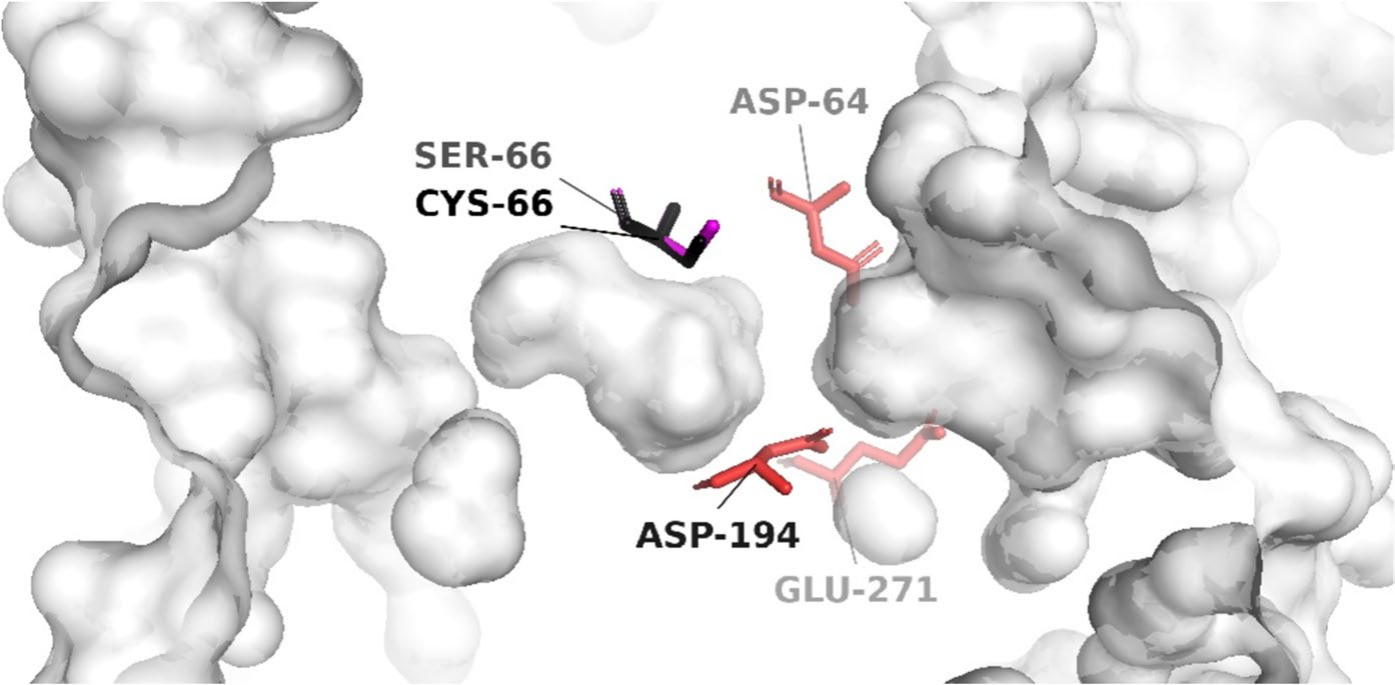
Superimposed tertiary structure alignments of C66S and SucC. The catalytic triad (D64, D194 and E271) were shaded in red and labelled. The Cys-66 and Ser-66 residues showed no differences in their conformational structures

The sucrose access tunnels for both the C66S mutant and wild-type SucC were predicted and depicted in Fig. 3. Two tunnels were modeled: tunnel one (T1—depicted in green) is proposed for substrate transport into the active site, whereas tunnel two (T2—shown in blue) facilitates the exit of products. It was observed that the bottleneck region of the T1 access tunnel in C66S was slightly reduced to 1.12 Å compared to 1.13 Å in the wild-type SucC, while the T2 bottleneck region remained unchanged at 0.71 Å. The length of the T1 tunnel for C66S was 18.90 Å, which was slightly longer than that of the wild-type SucC at 18.66 Å. Conversely, the T2 tunnel length was slightly reduced to 16.74 Å compared to 16.81 Å in the wild-type. Importantly, the curvature of both the T1 (1.23 Å) and T2 (1.15 Å) tunnels in C66S remained consistent with those in SucC. These findings demonstrate that substituting Cys with Ser does not result in significant changes to the conformational structure of the substrate access and product exit tunnels. Further structural analysis showed that the tertiary structure of the C66S mutant closely overlapped with that of the wild-type SucC, without noticeable conformational changes surrounding the active sites (Fig. 4).

In summary, the substitution of Cys-66 with Ser-66 is predicted to enhance the affinity between the substrate sucrose and the active sites by increasing hydrophilicity around the active site, without significantly altering the conformation of the active site.

### Biochemical characterization of mutant C66S

The C66S mutation in SucC was created using primer-mediated site-directed mutagenesis by the crossover PCR technique. The overlapped PCR fragment contained C66S mutation was subsequently cloned into pPIC9K. The resulting recombinant pPIC9K was then transformed into *P. pastoris* GS115 to obtain the correct recombinants. The recombinant enzymes SucC and C66S were prepared after shake flask fermentations and purified by size exclusion chromatography. The purified enzymes were used for further characterization.

### Specific activities

The specific activities of the purified C66S against sucrose were determined and are summarized in Table 2. Remarkably, mutant C66S had an increase of 61.3% in its specific activity compared to wild type SucC. The enzyme’s specific activity is a crucial parameter that reflects the efficiency of enzyme catalysis regarding per unit of protein mass (Xia et al. 2022). The increased specific activity indicates the C66S mutation significantly enhances the catalytic rate per enzyme molecule per unit time. This improvement implies that fewer C66S enzyme molecules are required to achieve the same reaction level. This may contribute to cost savings in FOS production processes.

**Table 2:** The specific activities of mutant C66S on catalysis of sucrose

Enzyme	Specific activity (U/mg)	Increased percentage (%)
SucC	10,159 ± 261	100
C66S	16,386 ± 405	161.3

### Kinetics parameters

The kinetics parameters of mutant C66S were examined and are summarized in Table 3. Mutant C66S presented a very similar *V*_max_ value (1.10 mM min^−1^) to that of SucC but a very different *K*_m_ value. The *K*_m_ value of C66S improved by 13.5% (71.14/82.21 mM) compared to that of SucC, indicating an increased affinity to sucrose in the catalysis. The *k*_cat_ value of C66S was increased by 21.6% (112.23/136.48 min^−1^) compared to that of SucC. As a result, the catalytic efficiency (*k*_cat_/*K*_m_) of C66S (1.92 min^−1^ mM^−1^) was increased by 1.4-fold to that of SucC (1.37 min^−1^ mM^−1^). The catalytic efficiency signifies the enzyme's effectiveness in converting substrates into products where a higher *k*_cat_/*K*_m_ value represents a greater catalytic efficiency of the enzyme (Nelson 2019). In this case, the 1.4-fold increase in catalytic efficiency of C66S to that of SucC indicated a more rapid conversion of sucrose into FOS under optimal conditions, contributing to an enhanced reaction speed. Improvements of the catalytic efficiency of fructosyltransferases is useful for industrial biocatalysts to influence the overall productivity and cost-effectiveness of processes (Guio et al. 2012; Kashyap et al. 2015). A comprehensive study on enhancing the catalytic efficiency of an α-amylase resulted in its improved kinetic properties for specific applications of interest (Hernández-Heredia et al. 2022). Moreover, the continuous effort to improve the catalytic efficiency not only optimizes existing enzymes but also provides valuable insights for enzyme engineering in which novel strategies and modifications are explored to enhance enzyme performance (Alvarado-Obando et al. 2022; Rakotoharisoa et al. 2023). Although there was a recent publication by Xia et al. (2022) on *A. niger* fructosyltransferase engineering to create the mutant C43S, the mutant showed reduced specific activity comparing to their wild type enzyme (545.4 compared to 2284.9 U mg^−1^). They focused on the mutant C43N and showed significant improvements in both catalytic efficiency (21.2-fold for *k*_cat_) and stability. In the current study, the analysis of mutant C66S correlated with the predictions and the enzyme kinetic studies showed that C66S did not have reduced enzyme performance compared to the wild type enzyme. The two studies therefore differed in the choice of the amino acid residue and the subsequent enzymatic data.

**Table 3:** The kinetic parameters of SucC and mutant C66S for catalysis of sucrose

Enzyme	*K*_m_ (mM)	*V*_max_ (mM^−1^ min^−1^)	*k*_cat_ (min^−1^)	*k*_cat_/*K*_m_ (min^−1^ mM^−1^)
SucC	82.20	1.10	112.23	1.37
C66S	71.14	1.11	136.48	1.92

### Temperature and pH optima, thermostability and pH tolerance

**Fig. 5:**
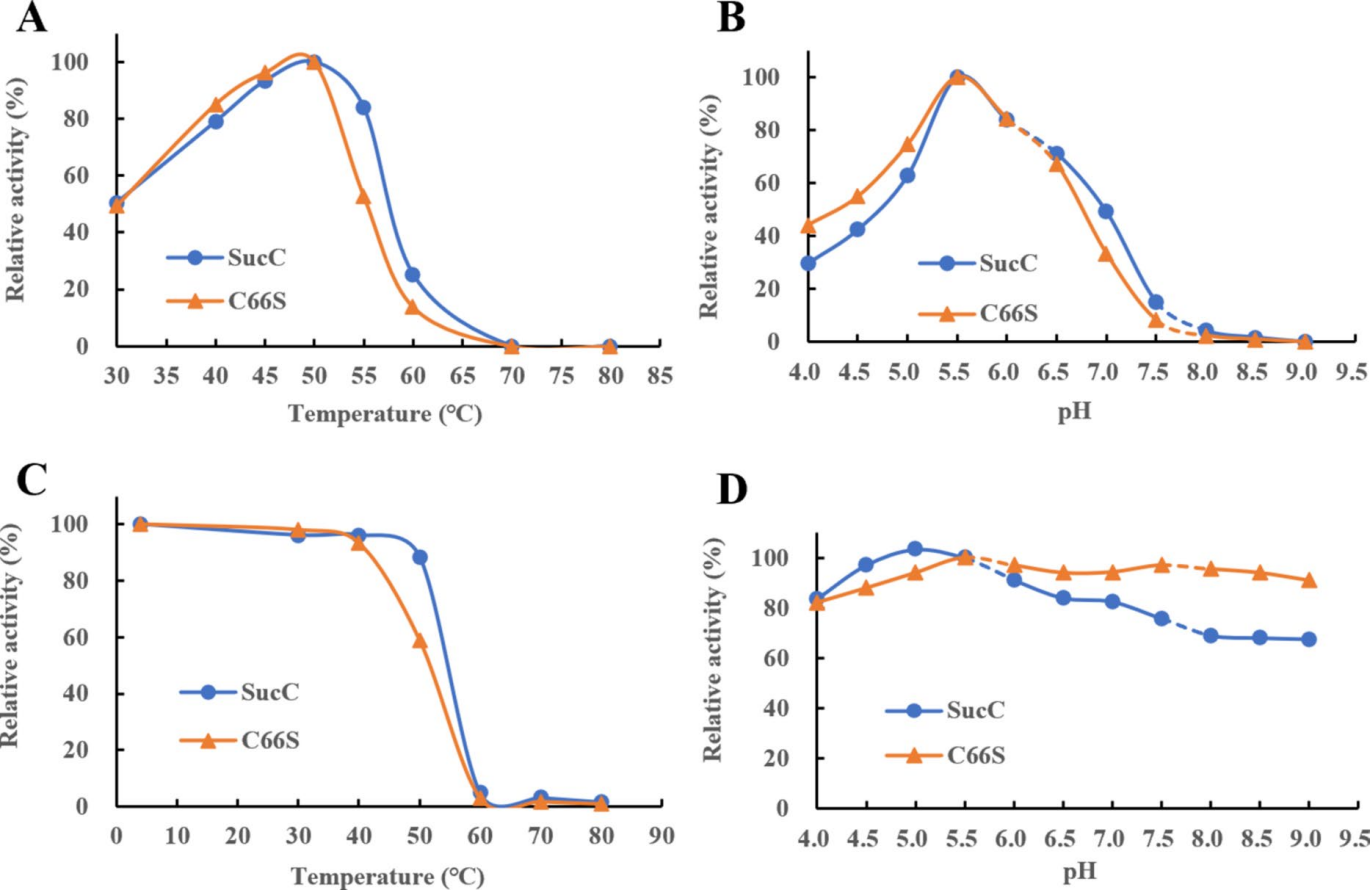
The effects of temperature and pH on the activities of SucC and mutant C66S. **A** Temperature optimum; **B** pH optimum; **C** thermostability at the temperatures from 4 to 80 ˚C for 1 h incubation; **D** pH stability at pHs from 4.0 to 9.0 for 1 h incubation

The optimal reaction temperature and pH of mutant C66S were determined and are summarized in Figs. 4a and 5b. The mutant C66S had maximum activity at 50 ˚C and pH 5.5, which are the same as that of SucC. The results indicated that the C66S mutation did not lead to a change of the temperature and pH optima.

The thermostability of C66S decreased compared to SucC (Fig. 4C). This suggested that the C66S mutation led to the change of the active site rigidity. The thermostability of an enzyme is affected by its tertiary structure conformation, salt bridge formed by charged residues, hydrogen bonds formed in the secondary and tertiary structure, the rigidity of amino acids and the hydrophobicity of protein (Nezhad et al. 2022). Typically, the disulfide (S–S) bridge formed by covalent linkages between Cys residues will greatly contribute to the stabilization of tertiary structure by improving the structural rigidity and hydrophobicity (Jiao et al. 2022). As a result, the substitution of Cys-66 to Ser-66 that created a more hydrophilic environment surrounding the catalytic nucleophile (Asp-64) that led to an increase in catalytic efficiency but a slight drop of thermostability due to the reduced rigidity of active sites where the -SH group is changed to -OH group. The pH stability was similar at the optimum pH (5.5) but SucC was more stable in the acidic environment and C66S was more stable in the alkaline environment (Fig. 4d). The reason for this is unclear.

### C66S mediated more efficient FOS production

**Fig. 6:**
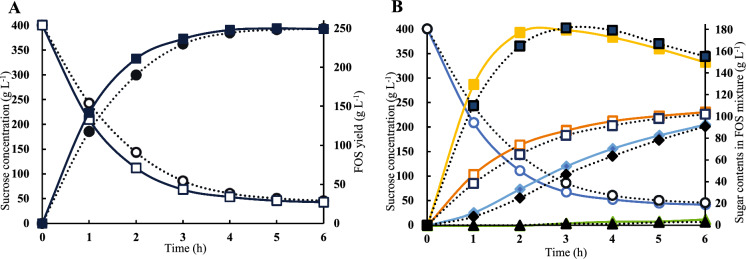
The time-course of FOS production from sucrose. The FOS preparation was carried out in 100 mL reaction volume with 400 g L^−1^ sucrose solution and 9 U g^−1^ enzyme at 50 ˚C and pH 5.5 for 6 h. The contents of sugars in the reaction mixture were determined by HPLC. **A** The residual sucrose (open rectangle or circle) and total FOS (solid rectangle or circle) catalysed by mutant C66S (solid line) or SucC (dotted line). **B** The sugar profiles (Circle: sucrose; Rectangle: glucose; Solid square: DP_3_; Solid diamond: DP_4_; Solid triangle: DP_5_) in the reaction mixture catalysed by C66S (solid lines) or SucC (dotted line)

Variant C66S-mediated FOS production from sucrose was conducted to further explore its catalytic performance and its potential for industrial application. The sugar profiles were analyzed by HPLC and the results are summarized in Fig. 6. Initially, 400 g sucrose L^−1^ was supplied for FOS production over 6 h. When the same amounts of enzymes (9 U g^−1^ sucrose) were supplied for the FOS production, an obvious increase in the reaction rate catalyzed by C66S was observed where the amount of sucrose consumption as well as the FOS (DP_3_, DP_4_, DP_5_) formation was more rapid than the reaction catalyzed by wild type SucC. C66S consumed 21.23% (residual 242.82/209.45 g sucrose L^−1^) more sucrose and produced 20.39% (117.48/141.44 g L^−1^) more FOS than the reaction catalyzed by wild SucC in the first hour, indicating a higher productivity in the early stages of FOS production. However, both reactions reached their endpoints by 4 h and the amount of sucrose consumption (355 g L^−1^) and FOS production (250 g L^−1^) were similar. These results validated the increased catalytic efficiency of C66S mediated a more efficient catalysis since *k*_*cat*_/*K*_*m*_ was higher compared with the wild type SucC but did not change its substrate specificity. The compositions of the FOS preparations were also analyzed (Fig. 6b). It was found that kestose (DP_3_), nystose (DP_4_) and fructofuranosylnystose (DP_5_) were the main components of FOS after 6 h of reaction. DP_3_ (150 g L^−1^) and DP_4_ (90 g L^−1^) were the main components of FOS while DP_5_ formation was only detected after 3 h for both reactions and at very low amounts (5 g L^−1^). Glucose (100 g L^−1^) was the main by-product. A low amount of fructose (< 5 g L^−1^) was also generated in both transfructosylating reactions (not shown). The overall FOS production catalyzed by C66S was more efficient than that catalyzed by wild type SucC, where the formation of DP_3_ was the most notable distinction between mutant C66S and wild type SucC where DP_3_ reached its maximum yield after 2 h when catalyzed by C66S and was faster than that catalyzed by wild type SucC (3 h). DP_3_ maximum yields for both enzymes were about 180 g L^−1^ and started to drop thereafter as DP_3_ served as the substrate to form DP_4_. However, the final spectrum of the generated FOS (DP_3_, DP_4_, DP_5_), glucose and fructose could not be distinguished between these two catalytic reactions, indicating no detectable changes in the FOS sugar profile using the C66S enzyme. The C66S enzyme had increased catalytic efficiency in transfructosylation without altering the enzyme’s substrate specificity. This observation further substantiated that the substitution of Cys-66 to Ser-66 in SucC led to an increased hydrophilic microenvironment surrounding the catalytic region, increasing its affinity to sucrose, and eventually improved its performance in catalytic efficiency during FOS production. The results of sugar profiles also demonstrated that the C66S variant was a competitively advantageous candidate for industrial production of FOS compared to the wild type SucC.

## Conclusion

In this study, the fructosyltransferase SucC from *A. niger* underwent targeted modifications to enhance its catalytic efficiency. By employing rapid artificial evolution techniques, including comprehensive structural analysis, simulated mutagenesis, and molecular docking, we successfully identified and constructed the C66S variant through site-directed mutagenesis. This variant enhanced the hydrophilicity around its active site, increasing substrate affinity and catalytic efficiency without significant structural alterations. Specifically, C66S demonstrated superior performance in producing FOS, particularly in the early stages of the reaction, compared to the wild-type SucC. The findings illustrate the potential of modifying the hydrophilic environment of enzyme active sites as a promising strategy for improving catalytic performance. These insights pave the way for future enzyme engineering efforts aimed at optimizing similar biocatalysts for industrial applications.

## Supplementary Information

Supplementary materials are attached as: Table S1. Summary of saturated mutagenesis Cys-66 showing of interacting residues and binding model by bioinformatic simulations. Fig S1. SDS-PAGE of AKTA purified SucC and C66S, indicating the pure enzymes obtained. and Fig S2. Partial presentation of DNA sequencing of C66S aligned with SucC, indicating the correct mutation of Cys-66 to Ser-66 (TGC to TCT).

## Supplementary Information

Below is the link to the electronic supplementary material.

Supplementary file1 (DOCX 1885 KB)

## Data Availability

The datasets generated and analyzed in the current study are available from the corresponding author on reasonable request.
