# Supplementary material for: Improving the hydrophilic microenvironment surrounding the catalytic site of fructosyltransferase enhances its catalytic ability
Source: Biotechnol Lett. 2025 Feb 26;47(2):30. doi: 10.1007/s10529-025-03566-8 (PMC11865173; doi:10.1007/s10529-025-03566-8)
Supplement: Supplementary file 1 — Supplementary file1 (DOCX 1885 KB) [file 10529_2025_3566_MOESM1_ESM.docx]

Table S 1 Summary of saturated mutagenesis Cys-66 showing of interacting residues and binding model by bioinformatic simulations

| **Enzyme** | **Binding energy (kcal/mol)** | **Binding residues** | **Surface model** |
| --- | --- | --- | --- |
| **SucC (WT)** | **-3.65** | **Asp^64^**  **Asp^122^**  **Arg^193^**  **Asp^194^**  **Glu^271^** | **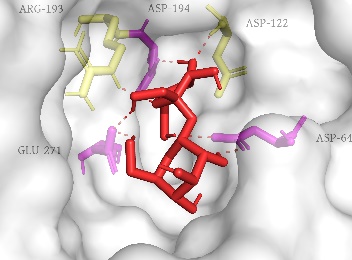** |
| **C66A** | **-3.81** | **Asp^64^**  **Asp^122^**  **Arg^193^**  **Asp^194^**  **Glu^271^** | **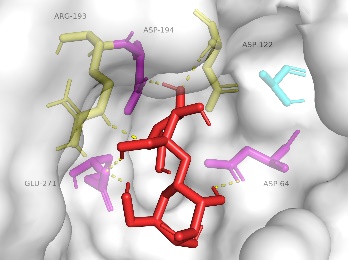** |
| **C66D** | **-3.88** | **Asp^64^**  **Asp^122^**  **Arg^193^**  **Asp^194^**  **Glu^271^** | **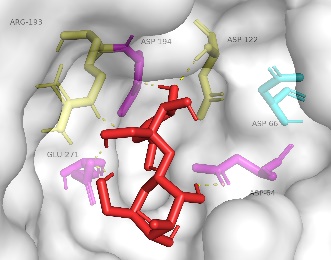** |
| **C66E** | **-3.76** | **Asp^64^**  **Asp^122^**  **Arg^193^**  **Asp^194^**  **Glu^271^** | **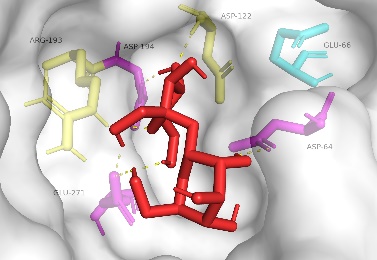** |
| **C66F** | **-3.66** | **Asp^64^**  **Asp^122^**  **Arg^193^**  **Asp^194^**  **Glu^271^** | **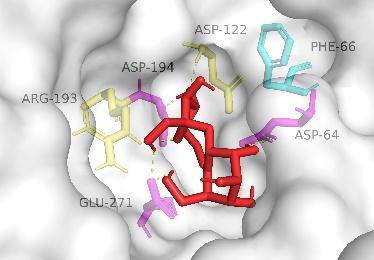** |
| **C66G** | **-3.86** | **Asp^64^**  **Asp^122^**  **Arg^193^**  **Asp^194^**  **Glu^271^** | **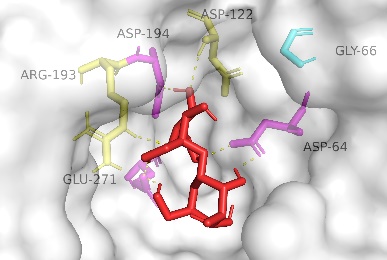** |
| **C66H** | **-3.54** | **Asp^64^**  **Asp^122^**  **Arg^193^**  **Asp^194^**  **Glu^271^** | **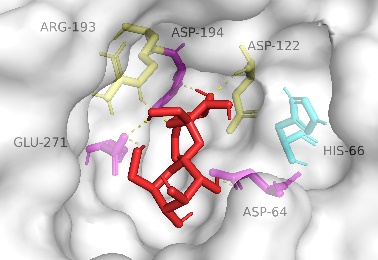** |
| **C66I** | **-3.7** | **Asp^64^**  **Asp^122^**  **Arg^193^**  **Asp^194^**  **Glu^271^** | **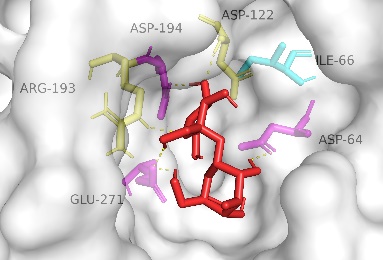** |
| **C66K** | **-3.07** | **Asp^64^**  **Asp^122^**  **Arg^193^**  **Asp^194^**  **Glu^271^** | **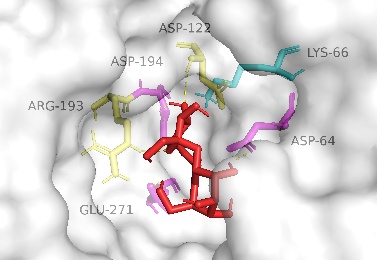** |
| **C66L** | **-3.72** | **Asp^64^**  **Asp^122^**  **Arg^193^**  **Asp^194^**  **Glu^271^** | **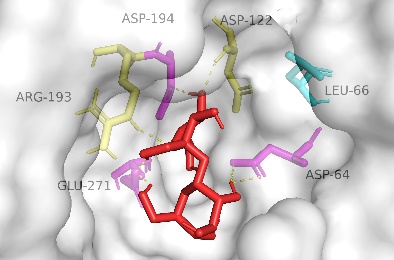** |
| **C66M** | **-3.32** | **Asp^64^**  **Asp^122^**  **Arg^193^**  **Asp^194^**  **Glu^271^** | **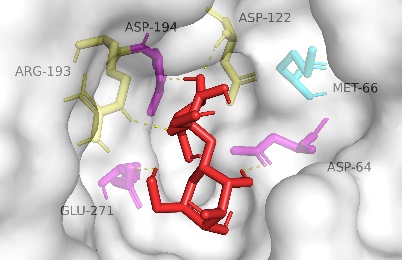** |
| **C66N** | **-3.85** | **Asp^64^**  **Asp^122^**  **Arg^193^**  **Asp^194^**  **Glu^271^** | **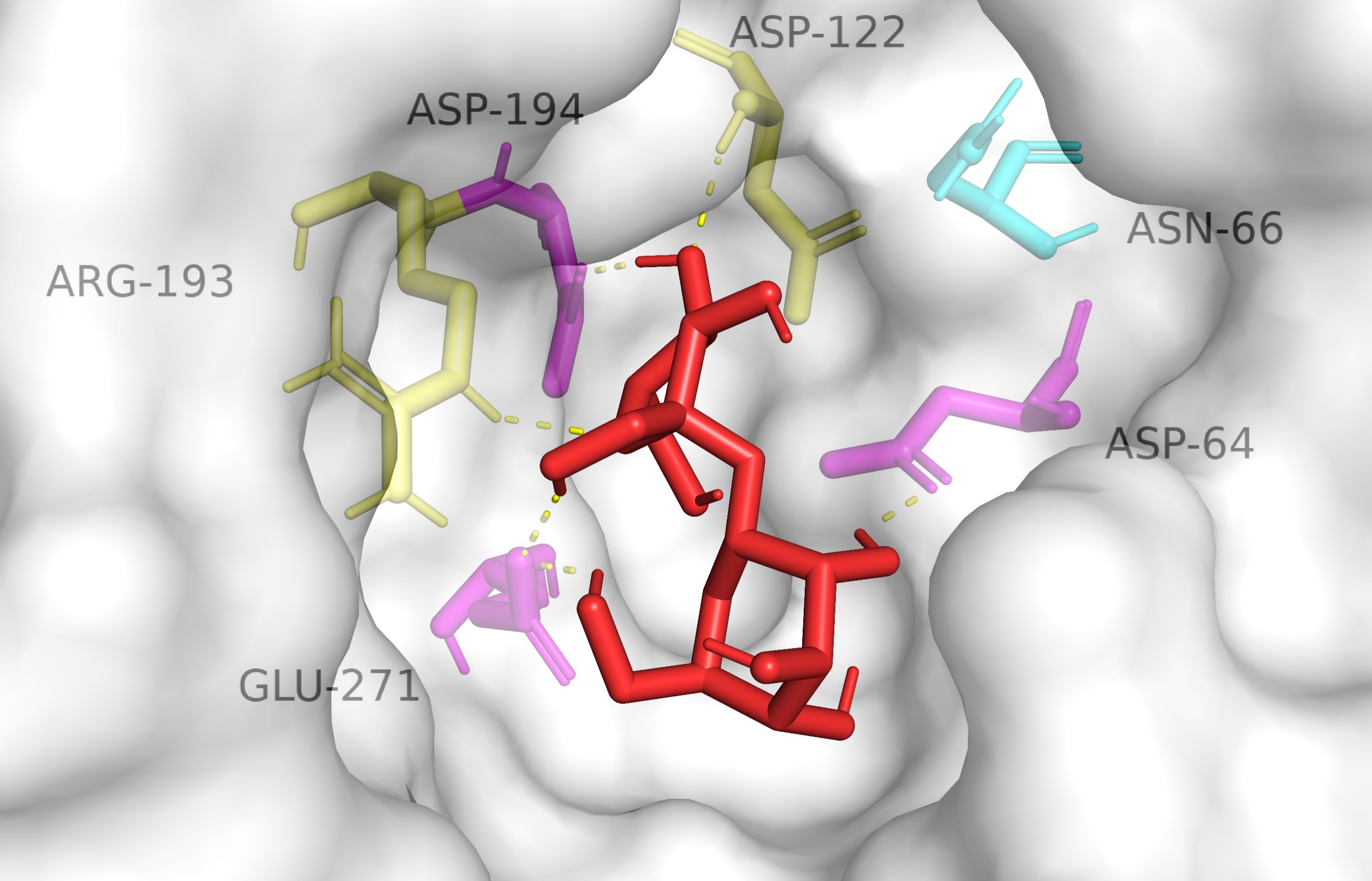** |
| **C66P** | **-3.86** | **Asp^64^**  **Asp^122^**  **Arg^193^**  **Asp^194^**  **Glu^271^** | **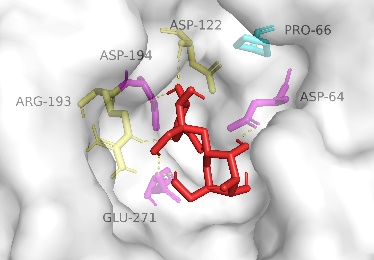** |
| **C66Q** | **-3.84** | **Asp^64^**  **Asp^122^**  **Arg^193^**  **Asp^194^**  **Glu^271^** | **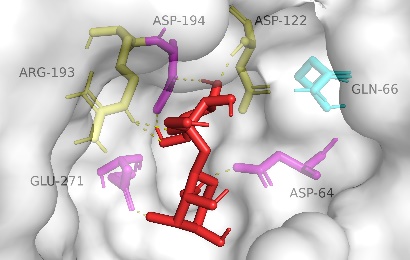** |
| **C66R** | **-3.58** | **Asp^64^**  **Asp^122^**  **Arg^193^**  **Asp^194^**  **Glu^271^** | **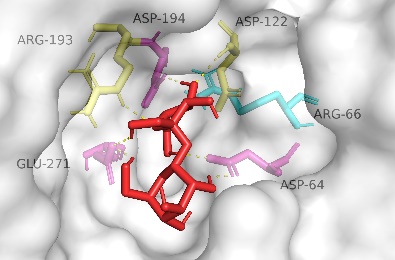** |
| **C66S** | **-4.14** | **Asp^64^**  **Asp^122^**  **Arg^193^**  **Asp^194^**  **Glu^271^**  **Glu^296^**  **His^310^** | **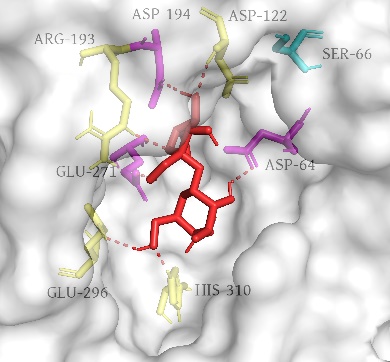** |
| **C66T** | **-3.81** | **Asp^64^**  **Asp^122^**  **Arg^193^**  **Asp^194^**  **Glu^271^** | **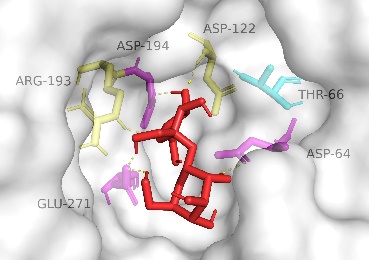** |
| **C66V** | **-3.86** | **Asp^64^**  **Asp^122^**  **Arg^193^**  **Asp^194^**  **Glu^271^** | **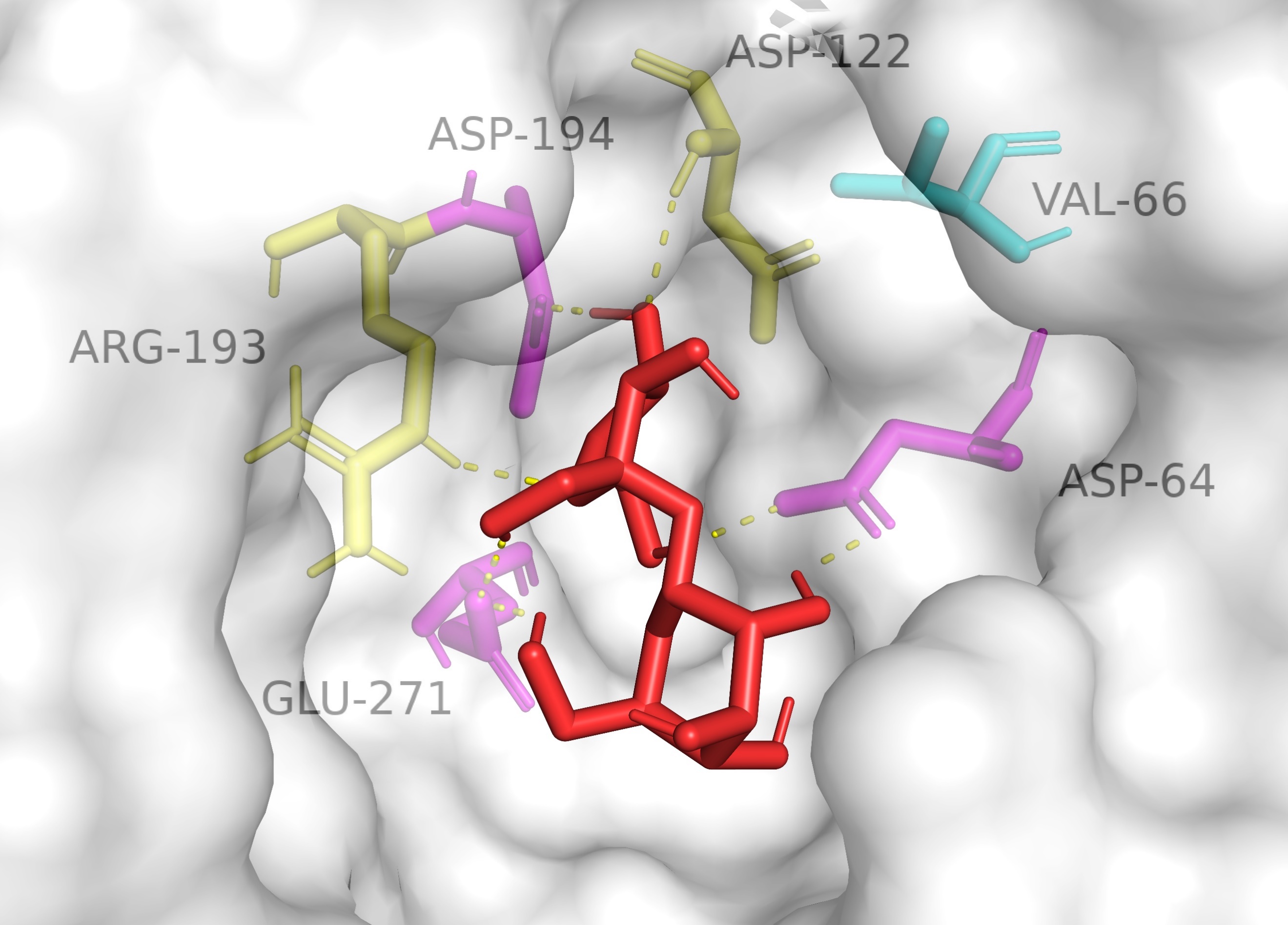** |
| **C66W** | **-3.84** | **Asp^64^**  **Asp^122^**  **Arg^193^**  **Asp^194^**  **Glu^271^** | **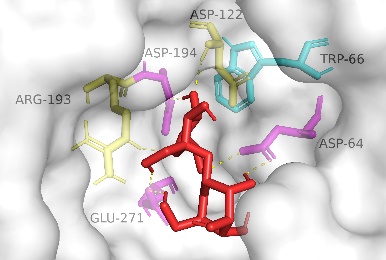** |
| **C66Y** | **-4.12** | **Asp^64^**  **Asp^122^**  **Arg^193^**  **Asp^194^**  **Glu^271^** | **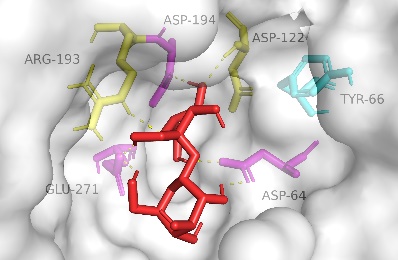** |


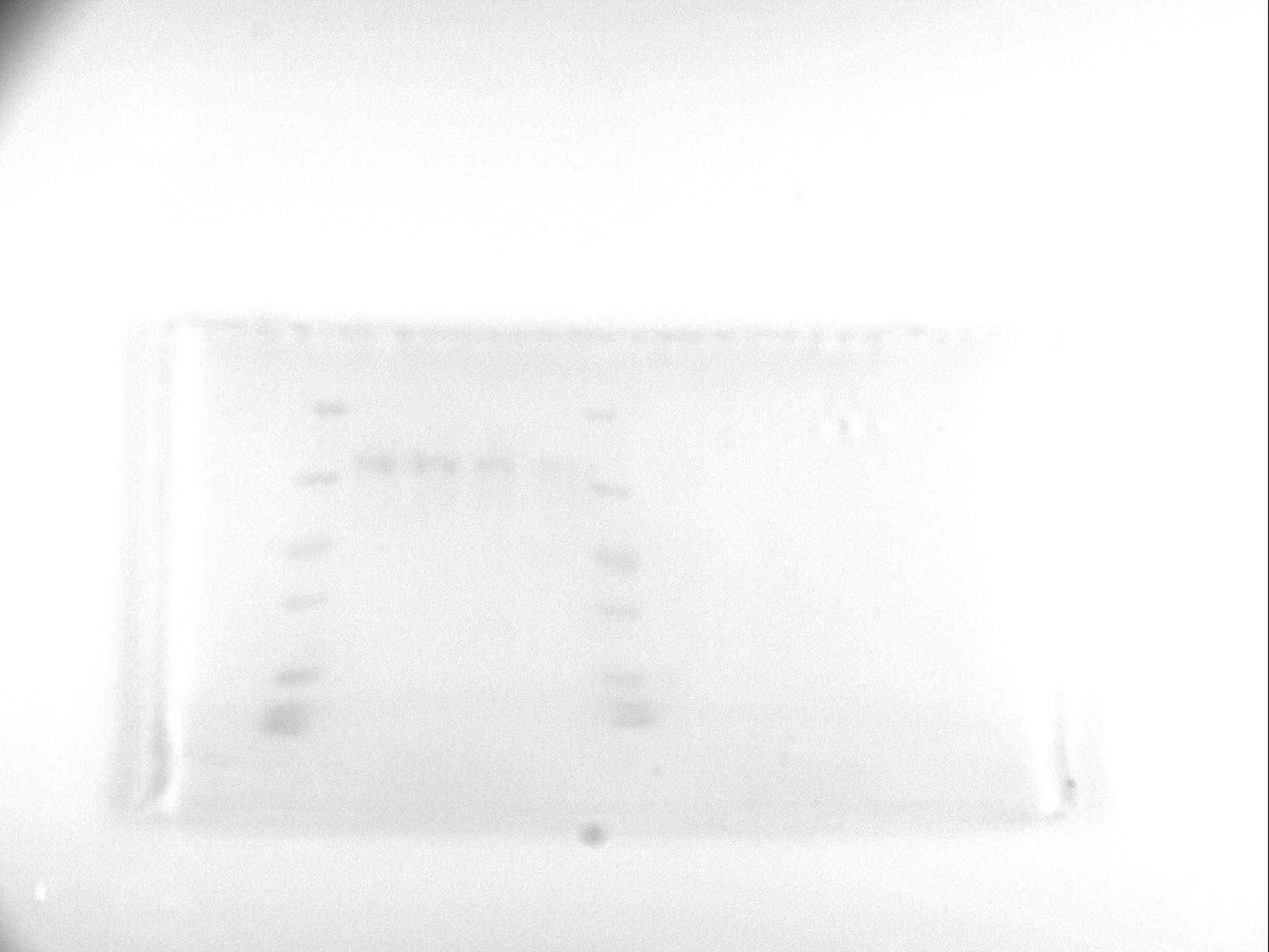
Fig. S 1. SDS-PAGE of AKTA purified SucC and C66S, indicating the pure enzymes obtained.

70.0 kDa

66.2 kDa

94.0 kDa

Fig. S 2. Partial presentation of DNA sequencing of C66S aligned with SucC, indicating the correct mutation of Cys-66 to Ser-66 (TGC to TCT).
